# Heart rate variability, sleep quality, and quality of life in epilepsy patients are multidependent and cannot be reduced to the quality of seizure control

**DOI:** 10.1590/1806-9282.20251895

**Published:** 2026-11-13

**Authors:** Sinda Zarrouk, Josef Finsterer

**Affiliations:** 1 University of Tunis El Manar and Genomic Platform, Pasteur Institute of Tunis – Tunis, Tunisia.; 2 Neurology and Neurophysiology Center, Department of Neurology – Vienna, Austria.

Dear Editor,

We read with interest the article by Duran et al. on a prospective cross-sectional study of the relationship between heart rate variability (HRV), quality of life, and sleep quality in 27 patients with drug-controlled epilepsy and 23 patients with drug-resistant epilepsy^1^. It was found that HRV was reduced in patients with epilepsy and that HRV correlated with cognitive function and well-being^1^. The study is interesting, but some points need to be discussed.

The first point is that subjective assessment of sleep quality using a questionnaire such as the Pittsburgh Sleep Quality Index may not adequately capture sleep quality. Only polysomnography can objectively assess whether sleep architecture is impaired or not. It is therefore recommended that the study be repeated using more sophisticated methods.

The second point is that sleep quality may depend not only on the quality of seizure control, but also on various other influencing factors. Numerous exogenous and endogenous factors also determine sleep quality and sleep hygiene^2^. These additional determinants of sleep quality must be taken into account when assessing the sleep performance of epilepsy patients and interpreting the study results. Adequate and restful sleep is a prerequisite for seizure control.

The third problem is that comorbidities were not taken into account as determinants of HRV, quality of life, and sleep quality^1^. Various conditions, such as myocardial disease, cardiac conduction disorders, alcoholism, obesity, psychiatric disorders, heat, noise, shift work, harmful and dangerous substances, orthopedic disorders, rheumatological disorders, immunological disorders, malignancies, and movement disorders can affect HRV, quality of life, and sleep^3^. Failure to consider comorbidities in the analysis could distort the results or even lead to false negative or false positive results.

The fourth point is that all antiseizure medications (ASMs) are associated with side effects^4^. Phenytoin and carbamazepine can reduce HRV^5^. Carbamazepine and phenobarbital impair sleep architecture^6^. Most ASMs reduce rapid eye movement sleep^6^.

The fifth point is that low HRV has been attributed to reduced parasympathetic tone^1^, suggesting that autonomic innervation is disrupted in epilepsy patients. Therefore, it is crucial to know how many of the included patients had polyneuropathy with autonomic involvement or pure autonomic neuropathy. Autonomic neuropathy can be caused by seizures that affect the central autonomic network or by the long-term effects of certain ASMs. Autonomic dysfunction may also be due to impairment of the central parasympathetic pathways.

In summary, all determinants of sleep quality, quality of life, and HRV must be considered when assessing their significance for epilepsy. As long as the interdependence of these parameters is not taken into account, their evaluation in epilepsy patients remains unclear.

## Data Availability

The datasets generated and/or analyzed during the current study are available from the corresponding author upon reasonable request.
